# Supplementary figures and images for: Dissimilar Friction Stir Welding of AA2519 and AA5182
Source: Materials (Basel). 2022 Dec 8;15(24):8776. doi: 10.3390/ma15248776 (PMC9786200; doi:10.3390/ma15248776)

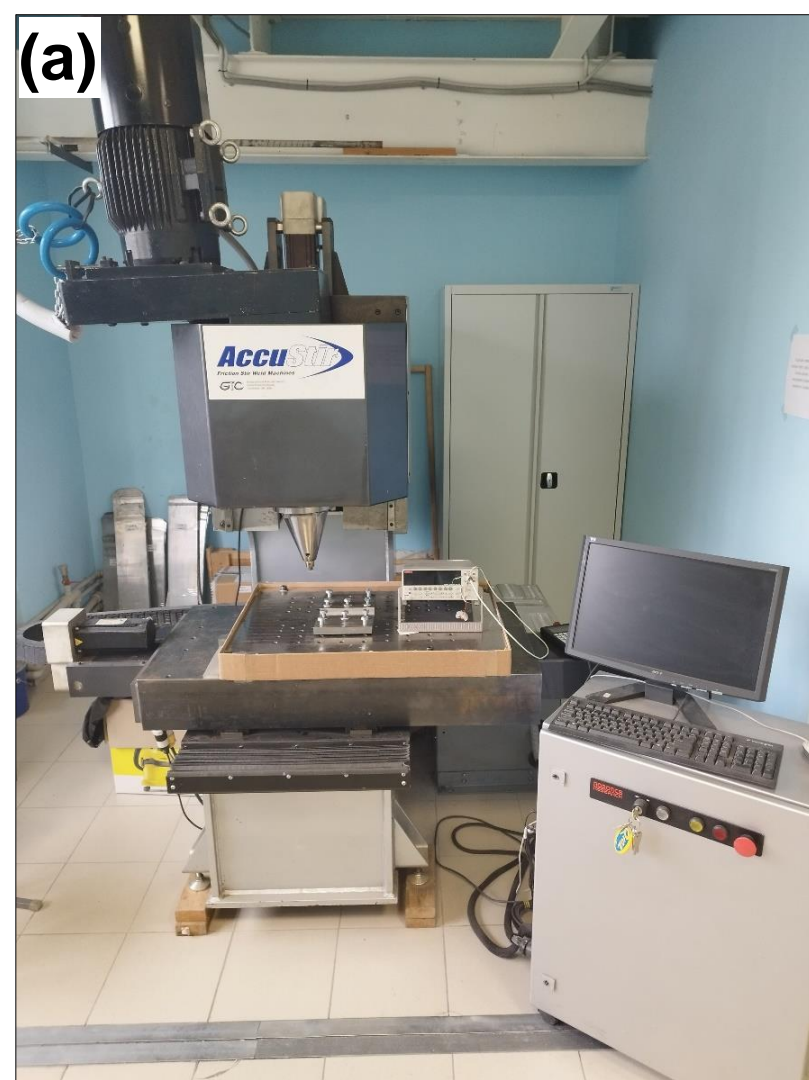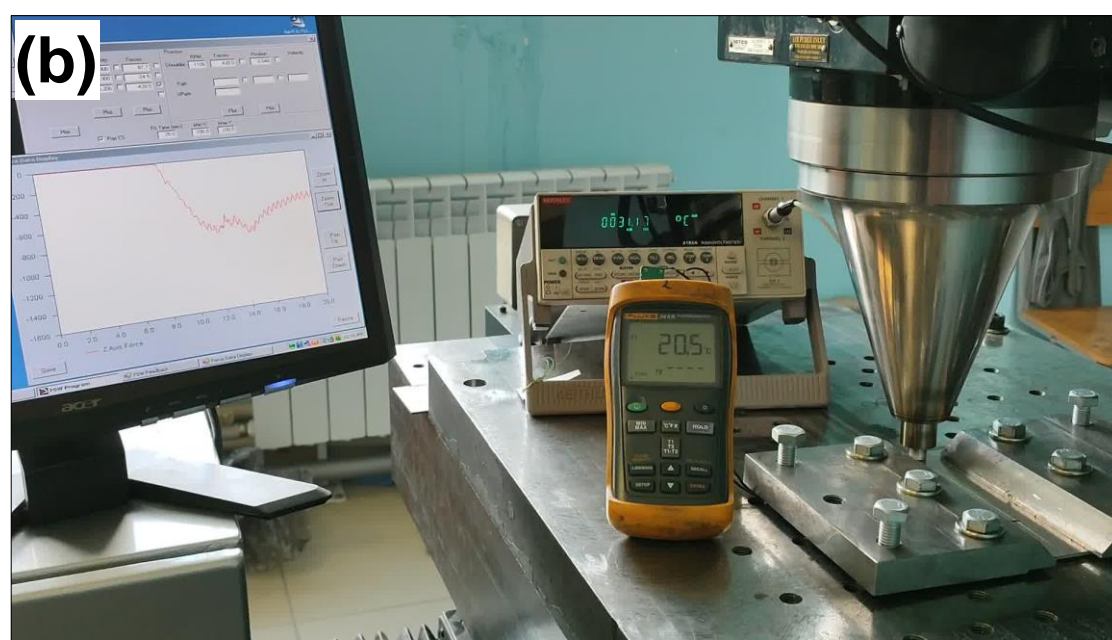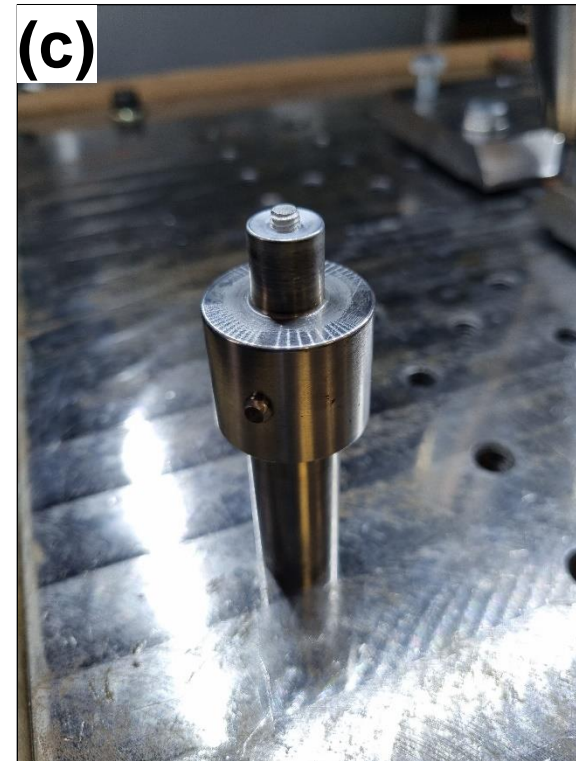

**Supplementary Figure S1.** Photographs of used FSW-machine (a), experiment setup (b) and tool (c).

Supplement: Supplementary file 1 [file materials-15-08776-s001.zip › materials-2051551-supplementary.pdf]
